# Structure-based design of a sequence-specific RNA probe that mimics the kink-turn motif

**DOI:** 10.1093/narmme/ugaf006

**Published:** 2025-03-18

**Authors:** Takumi Miyauchi, Kanna Yamaguchi, Satomi Saisu, Jiro Kondo

**Affiliations:** Department of Materials and Life Sciences, Faculty of Science and Technology, Sophia University, 7-1 Kioi-cho, Chiyoda-ku 102-8554 Tokyo, Japan; Department of Materials and Life Sciences, Faculty of Science and Technology, Sophia University, 7-1 Kioi-cho, Chiyoda-ku 102-8554 Tokyo, Japan; Department of Materials and Life Sciences, Faculty of Science and Technology, Sophia University, 7-1 Kioi-cho, Chiyoda-ku 102-8554 Tokyo, Japan; Department of Materials and Life Sciences, Faculty of Science and Technology, Sophia University, 7-1 Kioi-cho, Chiyoda-ku 102-8554 Tokyo, Japan

## Abstract

The technology for sequence-specific detection of RNA is in high demand in the medical field as well as in basic research in life sciences. Various methods for detecting RNA have been developed so far, but all of them are designed based solely on the rules of base complementarity, which leads to the false detection of unrelated RNAs with very similar sequences. In this study, we challenged the biomimetics approach at the molecular level to develop a sequence-specific RNA probe by mimicking a well-known RNA structural motif, the kink-turn motif, which exists in various functional RNAs. Our probe was designed in such a way that the formation of the kink-turn motif is induced only when it hybridizes with the target RNA, resulting in the exposure of the fluorescent base introduced into the probe. As we expected, both the RNA-based and DNA-based probes sensitively and selectively detected the target RNA as an increase in fluorescence intensity. We also confirmed the actuation mechanism of the probes by X-ray crystallography. This study showed that the RNA structural motifs remaining as a result of natural selection could be applied to biomimetics at the molecular level.

## Graphical Abstract

**Graphical Abstract:**
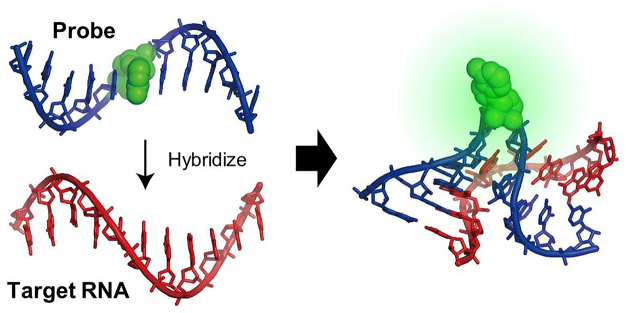

## Introduction

The technology for sequence-specific detection of RNA is in high demand in the medical field as well as in basic research in life sciences. SARS-Cov-2, which is currently experiencing a worldwide explosion of infection, the influenza virus, which rages around the world every year, and HIV, which causes severe immunodeficiency syndrome, have single-stranded RNA genomes. It has also been reported that specific microRNAs and long noncoding RNAs can be biomarkers for various cancers and other diseases [1–6]. Various techniques have been developed to detect these RNAs, ranging from inexpensive and simple ones such as northern blotting and *in situ* hybridization to more accurate and sensitive ones such as quantitative polymerase chain reaction (qPCR), microarray, and RNA-seq.

The molecular beacon is well-known as a simple and inexpensive RNA detection technique [7]. It is made of a DNA fragment, a fluorophore, and a quencher. The central part of the molecular beacon contains a sequence complementary to the target RNA, and the two ends have sequences complementary to each other. A fluorophore and a quencher are bound to the two ends of the molecular beacon, respectively. The complementary sequences at the two ends form a double helix, which brings the fluorophore and the quencher into proximity and prevents them from fluorescing. When the molecular beacon hybridizes with the target RNA using the sequence in its central part, the double helix formed between two ends unwinds, and the fluorophore and quencher separate, resulting in strong fluorescence. While the molecular beacon has such a simple actuation mechanism, it has several problems: (i) it requires both fluorophore and quencher, (ii) the lengths of the probe sequence in the center and complementary sequences placed at both ends must be optimized, and (iii) it may nonspecifically bind to and detect RNAs possessing similar sequences to the actual target RNA. Several methods, such as ECHO probes [8], have been developed to solve these problems but require special nucleotides.

**Figure 1.**
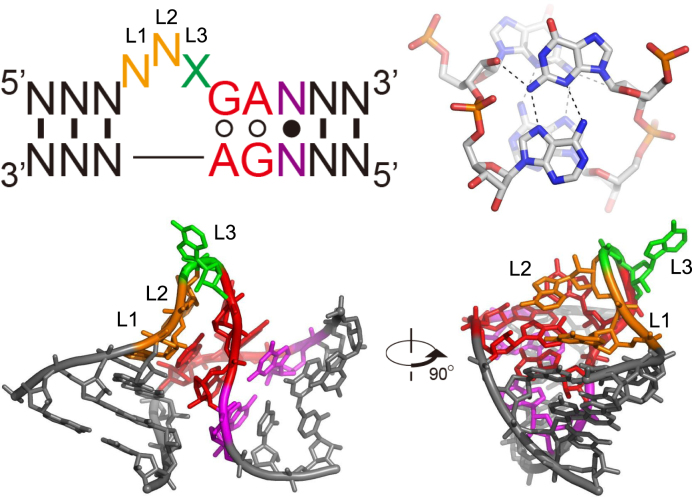
Secondary and tertiary (PDB ID: 4C40) structures of the kink-turn motif, and the sheared G-A base pair found in this motif. The two alternative sheared G-A base pairs and generally three bulged-out bases, named L1, L2, and L3, are essential for forming this structural motif. Base X at L3 position is fully exposed, and two bases N at position L1 and L2 are partially exposed. N-N indicates complementary base pairing, and N•N indicates complementary or noncomplementary base pairing.

**Figure 2.**
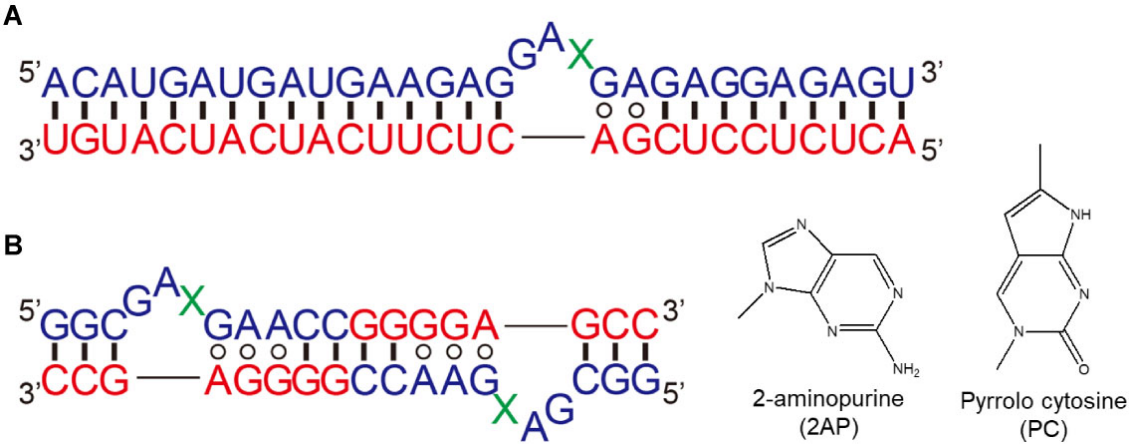
Oligonucleotides used in this study. Panel (**A**) shows a sequence-specific RNA probe designed by mimicking the kink-turn motif hybridized to the target RNA. Panel (**B**) shows the RNA (Kt-2AP or Kt-PC) used for X-ray crystallography. 2AP or PC was introduced at X. The circle indicates the formation of a noncomplementary sheared G-A base pair.

In this study, we challenged the biomimetics approach at the molecular level to develop a sequence-specific RNA probe that can be easily synthesized using commercially available nucleotides. We have focused on a well-known RNA structural motif, the kink-turn motif (Fig. 1), which exists in various functional RNAs such as ribosomal RNAs, riboswitches, small nuclear RNAs (snRNAs), small nucleolar ribonucleoproteins (snoRNPs), signal recognition particles, and ribonuclease P [9–11]. This motif contains two alternative sheared G-A base pairs and generally three bases not involved in base pair formation. As a result, the double-stranded RNA has a characteristic three-dimensional structure with a bend of ∼50°. It has been reported that various proteins bind to this characteristic structural motif and contribute to roles such as structural stabilization and translation regulation. Because of its functional and structural features, this structural motif has also been used as a building block in RNA nanotechnology [12–14]. Crystal structures of the kink-turn motif revealed by Lilley *et al.* [15] show that the X base at position L3 (Fig. 1) is fully exposed outside the molecule. In other words, replacing this X residue with a functional nucleotide would not affect the formation of the kink-turn motif. We first considered the shorter strand of the kink-turn motif as the target RNA and the longer strand, including the X residue, as the probe. We then designed a probe that can detect RNA in a sequence-specific manner by introducing a fluorescent base at X (Fig. 2A). If this probe hybridizes with the target RNA, the kink-turn motif should be formed, and the fluorescent base introduced at X might be exposed, which is expected to increase the fluorescence intensity. In this study, we evaluated the sequence-specific RNA detection ability of the designed probe and also confirmed its actuation mechanism by X-ray crystallography.

## Materials and methods

### Design, synthesis, and purification of oligonucleotides

The probe fragments composed of RNA or DNA nucleotides were designed to form a kink-turn motif when hybridized with the target RNA (Fig. 2). 2′-deoxyribonucleotide with a fluorescent base 2-aminopurine (2AP) or pyrrolo cytosine (PC) was introduced at X. The RNA-based probes containing 2AP or PC are called RNA–2AP and RNA–PC, respectively, and the DNA-based probe corresponding to RNA–2AP is called DNA–2AP.

The probe molecules, the target RNA, and five off-target RNAs (Table 1) were chemically synthesized using an automated nucleic acid synthesizer NTS-M2-MX (Nihon Techno Service). Phosphoramidites of nucleotides containing fluorescent bases, 2AP and PC, were purchased from Glen Research. The chemically synthesized samples were purified by gel filtration using NAP-10 columns (Cytiva). It was confirmed by 20% denaturing polyacrylamide gel electrophoresis containing 7 M urea that these oligonucleotides were synthesized correctly with high purity.

**Table 1.** Sequence of oligonucleotides used in this study

Oligonucleotides	Sequence
**Probes**	
RNA–2AP	5′-ACA UGA UGA UGA AGA GGA (2AP)GA GAG GAG AGU-3′
RNA–PC	5′-ACA UGA UGA UGA AGA GGA (PC)GA GAG GAG AGU-3′
DNA–2AP	5′-ACA TGA TGA TGA AGA GGA (2AP)GA GAG GAG AGT-3′
**Target RNA**	
Target RNA	5′-ACU CUC CUC GAC UCU UCA UCA UCA UGU-3′
**Off-target RNAs for probe design**	
Off-target RNA 1	5′-CGA GCG CCC CAC CAC CC-3′
Off-target RNA 2	5′-GGG CCG GUC CCC CGG G-3′
Off-target RNA 3	5′-CCC GGU UCU UGG CCG GCC C-3′
**Off-target RNAs for RNA detection experiment**	
Off-target RNA 4	5′-GCA ACA UCG GGU GAA GUC GGA GCC AUG-3′
Off-target RNA 5^a^	5′-ACU CUC CUC UCC UCU UCA UCA UCA UGU-3′

^a^In off-target RNA 5, substituted bases are underlined to indicate differences from the target RNA.

Using the RNA sequence crystallized by Lilley *et al.* for X-ray analysis of the Kink-turn motif [15] as a reference, sequences introducing 2AP or PC at the sixth X residue (Kt-2AP and Kt-PC) were designed and chemically synthesized (Fig. 2). These oligonucleotides were purified by 20% denaturing polyacrylamide gel electrophoresis containing 7 M urea (30 cm × 40 cm size) and then desalted by gel filtration using NAP-10 columns.

### Fluorescence spectra measurement for probe design

Fluorescence spectra measurements for designing the probe molecules were conducted using FP-8300 with the single-drop measurement unit (Jasco). First, 3D fluorescence spectra of a control solution (10 μl) containing 0.01 mM probe fragment (RNA–2AP, RNA–PC, or DNA–2AP), 10 mM sodium cacodylate, and 100 mM sodium chloride were measured to determine the maximum excitation and fluorescence wavelengths of the probe fragment. Next, solutions containing 0.01 mM probe fragment and 0.01 mM target or off-target RNA were prepared in 10 mM sodium cacodylate and 100 mM sodium chloride, and their fluorescence spectra were measured. The ability of the probe fragments to detect the target RNA was evaluated by calculating the fluorescence intensity change rate (*ΔF*) using the following equation, where *F*_0_ is the fluorescence intensity of the probe alone, and *F* is the fluorescence intensity after the addition of the target or off-target RNA.


\begin{eqnarray*}
\Delta F = \left( {F - {{F}_0}} \right)/{{F}_0}
\end{eqnarray*}

### RNA detection experiments using the DNA-based probe

In contrast, fluorescence measurements for practical RNA detection applications using the DNA–2AP probe were performed with a microplate reader, Varioskan LUX (Thermo Fisher Scientific) to accommodate high-throughput sample processing. Since the microplate format requires a larger sample volume (∼100 μl) compared to the single-drop measurement unit of the FP-8300 (∼10 μl), the total reaction volume was increased 10-fold. To minimize reagent costs while maintaining detection sensitivity, the probe concentration was adjusted to the lower detection limit of the microplate reader. As a result, the DNA–2AP probe concentration in these experiments (0.001 mM) was reduced to one-tenth of that used in the fluorescence spectra measurements mentioned in the previous section. For each measurement, 100 μl of solutions containing 0.001 mM DNA–2AP probe, 0.001 mM target or off-target RNA, 10 mM sodium cacodylate, 100 mM sodium chloride, and 200 mM magnesium chloride was prepared in a 96-well, black, V-bottom plate. The fluorescence intensity at 370 nm was recorded for each condition.

### Dependence of fluorescence intensity on target RNA concentration

To examine how fluorescence intensity changes with increasing target RNA concentration, we performed titration experiments using the DNA–2AP probe. The fluorescence intensity of the DNA–2AP probe (0.01 mM) was measured under varying target RNA concentrations, with [Target RNA]/[DNA–2AP] ratios of 0.1, 0.2, 0.3, 0.4, 0.5, 0.6, 0.7, 0.8, 0.9, 1.0, 1.1, 1.2, 1.3, 1.4, 1.5, and 2.0. These measurements were conducted using FP-8300 (Jasco) with the single-drop measurement unit under the same buffer conditions as the fluorescence spectra measurements for probe design.

### X-ray crystallography

Crystallizations were performed by the hanging-drop vapor diffusion method. Crystallization droplets were prepared by mixing 0.2 μl of 1 mM RNA (Kt-2AP or Kt-PC) with 0.2 μl of crystallization buffer. The droplets were equilibrated against a reservoir solution of 40% 2-methyl-2,4-pentandiol. Single crystals were obtained in a few days under the conditions shown in Table 2. Prior to X-ray diffraction experiments, the single crystals were scooped up with a CryoLoop (Hampton Research) and then frozen in liquid nitrogen.

**Table 2.** Crystallization conditions

Crystal code	Kt-2AP-1	Kt-2AP-2	Kt-PC-1	Kt-PC-2
**Sample solution: 0.2 μl**				
RNA	1 mM Kt-2AP	1 mM Kt-2AP	1 mM Kt-PC	1 mM Kt-PC
**Crystallization solution: 0.2 μl**				
Sodium cacodylate (pH 7.0)	50 mM	50 mM	50 mM	50 mM
Hexammine cobalt chloride	10 mM	–	–	–
Spermine tetrahydrochloride	–	10 mM	10 mM	10 mM
Strontium chloride	100 mM		100 mM	–
Potassium chloride	–	100 mM	–	100 mM
2-Methyl-2,4-pentandiol	10%	10%	10%	10%
**Reservoir solution: 250 μl**				
2-Methyl-2,4-pentandiol	40%	40%	40%	40%

X-ray diffraction experiments were carried out in the Structural Biology Beamlines BL-5A and BL-17A at Photon Factory (Tsukuba, Japan). The program *XDS* [16] was used to process the diffraction data. The initial phases were determined by the molecular replacement method using the program *Phaser* in the *Phenix* suite [17–19]. A crystal structure of the kink-turn motif solved by Lilley *et al.* (PDB ID: 4C40) was used as a molecular replacement probe [15]. Molecular models were constructed using the program *Coot* [20, 21]. The atomic parameters were refined using the program *phenix.refine* in the *Phenix* suite [18, 19]. The crystal structures solved in this study were deposited in the Protein Data Bank (PDB ID: 7EI7, 7F8Z, 7EI9, and 7EIA).

## Results and discussions

### Detection of the target RNA by the probe made of RNA

Although our ultimate goal is to develop a DNA-based probe due to its higher stability and cost-effectiveness compared to RNA, we first sought to confirm whether a kink-turn-mimicking probe could be designed successfully. To evaluate the feasibility of our approach, we investigated the ability of the RNA–2AP probe to detect the target RNA in a sequence-specific manner by measuring its fluorescence spectrum.

**Figure 3.**
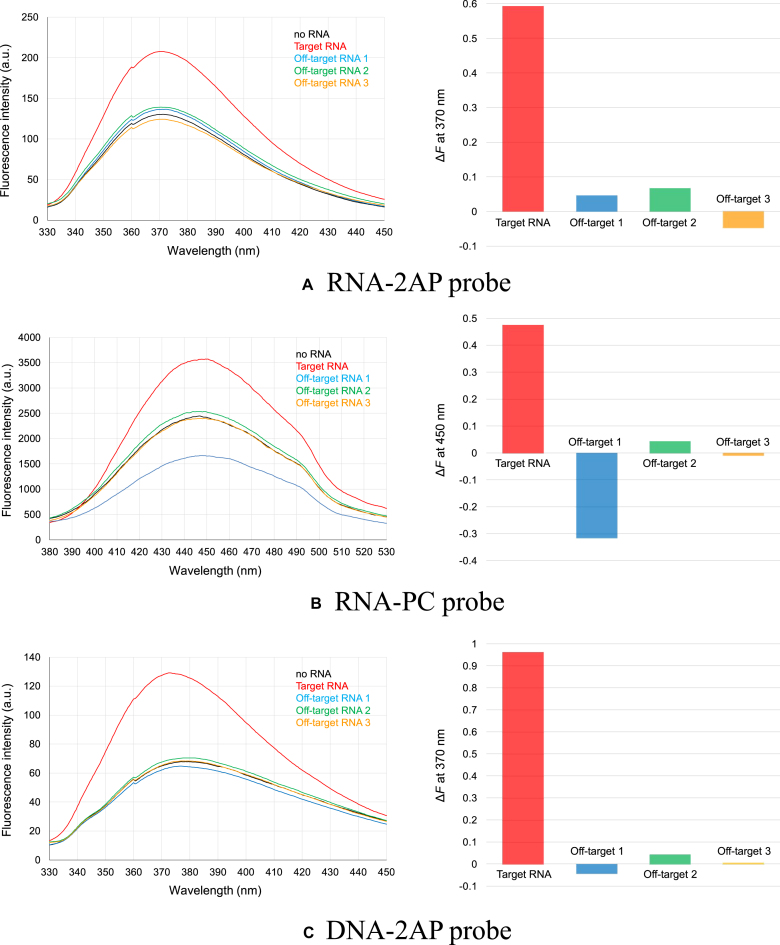
Sequence-specific RNA detection ability of the RNA–2AP (**A**), RNA–PC (**B**), and DNA–2AP (**C**) probes. The left figures show fluorescence spectra in the absence and presence of the target RNA or off-target RNAs; the right figures show the fluorescence intensity change rate *ΔF*.

The fluorescence spectra of the RNA–2AP probe in the range of 330–450 nm were measured under an excitation wavelength of 305 nm (Fig. 3A). The fluorescence intensity of the RNA–2AP probe remained almost unchanged when any of the three off-target RNAs were added. In contrast, when the target RNA was added, the fluorescence intensity of the RNA–2AP probe increased significantly. The fluorescence intensity change rate (*ΔF*) for the target RNA (0.59) was notably higher than any of the three off-target RNAs (−0.05–0.07). These results confirm that the RNA–2AP probe can detect the target RNA in a sequence-specific manner, validating our design strategy.

### Effect of the type of fluorescent base

Before developing the DNA-based probe, we also examined whether different types of fluorescent bases could be incorporated at X of the probe fragment (Fig. 2A) without disrupting its function. To investigate this, we performed the same experiment described above using the RNA-PC with PC instead of 2AP.

The fluorescence spectra were measured in the range of 380–530 nm under an excitation wavelength of 270 nm (Fig. 3B). Similar to the RNA–2AP probe, the fluorescence intensity of the RNA–PC probe significantly increased only when the target RNA was added (Δ*F* = 0.47). However, in contrast, when off-target RNA 1 was introduced, the fluorescence intensity of the RNA–PC probe decreased significantly (Δ*F* = −0.32).

To understand the reason for this fluorescence quenching, we estimated whether the RNA–PC probe could hybridize with the off-target RNA 1 using the program RNAstructure [22]. The result of secondary structure prediction indicates that the sequence 5′-GGA(PC)G-3′ in the RNA–PC probe can hybridize with the sequence 5′-CGUCC-3′ in the off-target RNA-1 by forming Watson–Crick base pairs (Supplementary Fig. S1). Since PC can form the Watson–Crick type base pair with G, PC likely hides within the double helix, and as a result, a decrease in fluorescence intensity was observed. In fact, the same phenomenon has been applied to RNA sequence-specific detection in a previous study [23].

These results demonstrate that different fluorescent bases with varying excitation and emission wavelengths can be introduced at X of the probe fragment, depending on the intended application. Importantly, this validation enabled us to refine the design before constructing the DNA-based probe, which is described in the following section.

### Detection of the target RNA by the probe made of DNA

The kink-turn motif is an RNA structural motif found in several functional RNA molecules. Therefore, it was expected that the kink-turn motif would be formed when an RNA-based probe hybridized with the target RNA. However, for practical applications, DNA is a more suitable choice due to its higher chemical stability and lower cost compared to RNA. Thus, we aimed to determine whether a DNA-based probe could also mimic the kink-turn motif and function as effectively as the RNA-based counterpart. To investigate this, fluorescence measurements were first performed using the FP-8300 spectrofluorometer under the same conditions as those used in the RNA-based probe experiments.

The fluorescence spectra of the DNA–2AP were measured in the range of 330–450 nm with an excitation wavelength of 305 nm (Fig. 3C). The results demonstrated a significant increase in fluorescence intensity only when the target RNA was added, as observed in the RNA–2AP probe experiments. Moreover, the fluorescence intensity change rate (Δ*F* = 0.96) was even higher than that of the RNA-based probe (Δ*F* = 0.59). These findings strongly suggest that the DNA-based probe successfully hybridizes with the target RNA and induces a structural change similar to the RNA-based probe. DNA typically adopts a B-form helix when hybridizing with another DNA strand, whereas it adopts an A-form helix when hybridizing with RNA [24]. This general structural rule suggests that the DNA-based probe can induce the formation of the kink-turn motif when hybridized with the target RNA.

### Expanded validation using a microplate reader and additional off-target RNAs

**Figure 4.**
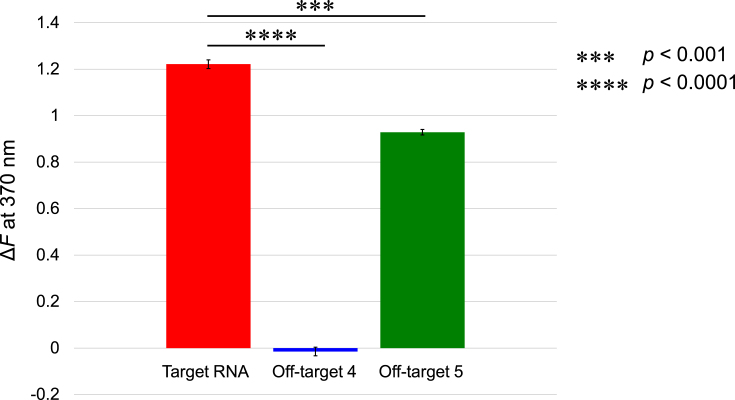
RNA detection experiments using the DNA–2AP probe. The figure shows the fluorescence intensity change rate *ΔF* at 370 nm in the presence of the target RNA, off-target RNA 4, and off-target RNA 5. Data are presented as mean ± standard error of the mean (SEM), with *n* = 3 independent measurements. Statistical significance was determined using an unpaired Student’s *t*-test. Asterisks indicate significant differences.

To further refine the evaluation of the DNA–2AP probe, we investigated its specificity by introducing two additional off-target RNAs. One of them, off-target RNA 4, was designed to have the same length as the target RNA while differing in sequence. As expected, the fluorescence intensity remained unchanged upon its addition (Fig. 4). In contrast, off-target RNA 5 contained two mutations that replaced the sheared G-A/A-G base pairs essential for kink-turn formation with Watson–Crick G-C/A-U base pairs. When this off-target RNA was introduced, fluorescence intensity increased, though to a lesser extent than when the target RNA was added (Fig. 4). This result aligns with our design expectations, as hybridization between DNA–2AP and off-target RNA 5 likely induced a structural change in which the 2AP base, along with adjacent bases, was incorporated into a bulge, forming either a 5′-GA(2AP)-3′ or 5′-A(2AP)G-3′ motif. In such cases, 2AP is expected to engage in stacking interactions with neighboring bases, which can lead to fluorescence quenching. Consequently, fluorescence enhancement was less pronounced compared to that observed with the target RNA.

These findings demonstrate that the DNA–2AP probe efficiently detects the target RNA through kink-turn motif formation, confirming its viability as a DNA-based probe. Additionally, the fluorescence measurements performed using a microplate reader establish the applicability of this approach for high-throughput RNA screening, reinforcing its potential for large-scale RNA detection.

### Concentration-dependent detection of the target RNA by the probe

**Figure 5.**
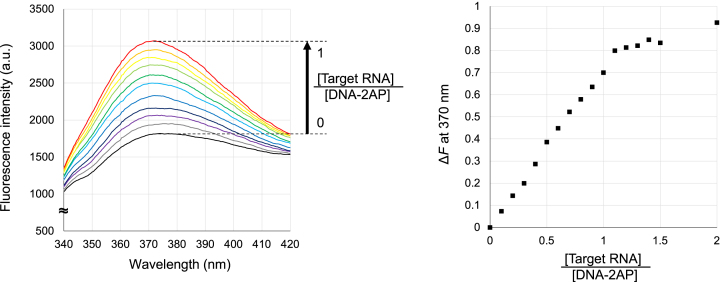
Concentration-dependent detection of the target RNA by the DNA–2AP probe. The left figure shows the fluorescence spectra under the condition of [Target RNA]/[DNA–2AP] from 0 to 1, and the right figure shows the plot of the fluorescence intensity change rate Δ*F* under the condition of [Target RNA]/[DNA–2AP] from 0 to 2.

To evaluate the ability of the DNA–2AP probe to detect target RNA in a concentration-dependent manner, the fluorescence spectra were recorded in the range of 340–420 nm under an excitation wavelength of 305 nm (Fig. 5). The fluorescence intensity of the DNA–2AP probe increased as the concentration of the target RNA increased. The fluorescence intensity reached its maximum when the concentration ratio of the probe to the target RNA was ∼1:1, after which no further increase in fluorescence was observed with additional target RNA. These results indicate that the probe detects the target RNA in a concentration-dependent manner, with saturation occurring at equimolar conditions. Furthermore, the results provide insights into the probe’s dynamic range and highlight its potential applicability for quantitative RNA detection.

### Confirming the actuation mechanism of the RNA probe by X-ray crystallography

We performed X-ray analyses to confirm whether our probes hybridize to the target RNA and induce the formation of the kink-turn motif, resulting in the fluorescent base’s exposure as designed. The RNA fragments constructed for the structural study, Kt-2AP and Kt-PC, were successfully crystallized in two different crystal forms (Table 2), and their structures were solved (Supplementary Fig. S2). The crystallographic data and statistics of data collections and structure refinements are summarized in Table 3.

**Table 3.** Crystal data, and statistics of data collections and structure refinements

Crystal code	Kt-2AP-1	Kt-2AP-2	Kt-PC-1	Kt-PC-2
PDB ID	7EI7	7F8Z	7EI9	7EIA
**Crystal data**				
Space group	*P*4_2_2_1_2	*P*2_1_2_1_2	*P*4_2_2_1_2	*P*222_1_
Unit cell (Å)	*a* = *b* = 75.9 *c* = 41.2	*a* = 66.0 *b* = 87.7 *c* = 124.3	*a* = *b* = 76.4 *c* = 41.2	*a* = 69.1 *b* = 77.6 *c* = 81.0
**Data collection**				
Beamline	BL-5A of PF	BL-17A of PF	BL-17A of PF	BL-17A of PF
Wavelength (Å)	1.60 496	0.98	0.98	0.98
Resolution (Å)	38.0–3.2	35.0–3.0	36.3–2.7	43.5–3.0
Of the outer shell (Å)	3.3–3.2	3.1–3.0	2.8–2.7	3.1–3.0
Unique reflections	2201	14 856	3622	8773
Completeness (%)	100.0	98.7	99.3	95.7
In the outer shell (%)	100.0	99.2	100.0	95.8
*R*_merge_^a^ (%)	6.6	9.9	3.6	4.0
In the outer shell (%)	44.8	39.6	39.7	38.3
Redundancy	23.2	5.8	4.1	3.4
In the outer shell	24.6	5.9	4.4	3.3
*I*/σ(*I*)	39.6	14.7	22.8	18.3
In the outer shell	8.8	5.2	3.6	2.7
**Structure refinement**				
Resolution range (Å)	38.0–3.2	35.0–3.0	36.3–2.8	43.5–3.0
Used reflections	2194	14 856	3619	8739
*R*-factor ^b^ (%)	24.5	17.6	22.7	20.0
*R*_free_^c^ (%)	30.5	22.9	24.6	26.8
R.m.s.d. bond length (Å)	0.007	0.006	0.007	0.007
R.m.s.d. bond angles (°)	1.2	1.1	1.1	1.2

^a^*R*_merge_ = 100 × Σ*_hklj_*|*I_hklj_* – <*I_hklj_* >| / Σ*_hklj_*<*I_hklj_*>.

^b^*R*-factor = 100 × Σ||*F*_o_| – |*F*_c_|| / Σ|*F*_o_|, where |*F*_o_| and |*F*_c_| are optimally scaled observed and calculated structure factor amplitudes, respectively.

^c^Calculated using a random set containing 10% of observations.

**Figure 6.**
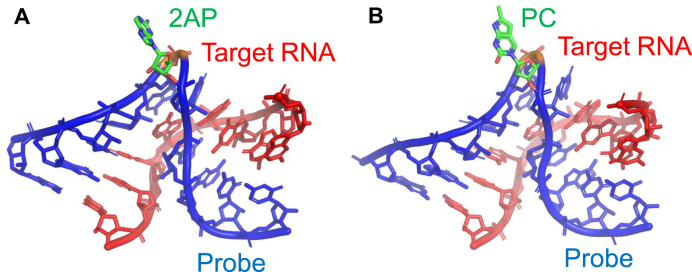
Structures of the kink-turn motif observed in Kt-2AP (**A**) and Kt-PC (**B**). Fluorescent bases 2AP and PC are fully bulged out to the solvent region.

The obtained structures revealed that the RNA adopted the canonical kink-turn conformation, which is entirely consistent with previously reported kink-turn structures, including those solved by Lilley *et al.* (Supplementary Fig. S3). Superposition of the crystal structures of Kt-2AP and Kt-PC onto the reference kink-turn structure (PDB ID: 4C40) [15] confirmed their structural similarity. The RMSD values for all atoms were 0.9 Å for Kt-2AP and 0.8 Å for Kt-PC. The characteristic asymmetric internal loop, consisting of sheared G-A/A-G base pairs, induces a sharp bend in the RNA backbone, creating the distinctive kink-turn geometry. As expected, 2AP and PC introduced in the sequence were largely exposed from the RNA backbone (Fig. 6 and Supplementary Fig. S3). On the other hand, two other bulged bases at positions L1 and L2 are stacked on the canonical helix (C helix) and the noncanonical helix (NC helix), respectively. This observation provides direct structural validation that 2AP and PC reliably bulge out when introduced at position L3. Specifically, the bulging 2AP and PC are positioned on the convex side of the kink, away from the helical stack, where they remain solvent-accessible. This exposure is crucial for fluorescence-based detection, as it ensures that the fluorophore is not engaged in extensive base stacking or hydrogen bonding, which could otherwise lead to fluorescence quenching.

Since our probe design was based on the assumption that X at the L3 position would be exposed upon kink-turn formation (Fig. 1), this structural confirmation strongly supports the results obtained in our RNA-based fluorescence experiments. The overall consistency of the structure with known kink-turn motifs further reinforces the robustness of our design, demonstrating that the kink-turn scaffold can be effectively utilized as a platform for fluorescence-based RNA detection.

One key structural feature of kink-turn motifs is the involvement of 2′-OH groups in stabilizing the sharp kink, as demonstrated in previous studies by Lilley *et al.* [25]. In particular, the 2′-OH group in the position L1 (Fig. 1), the 5′-most nucleotide within the bulge region of the motif, plays a critical role by forming hydrogen bonds that reinforce the sharp bending of the motif. This interaction is thought to contribute to the overall stability of the kink-turn architecture by assisting the stacking of adjacent bases and facilitating proper backbone conformation. Given this well-established role of 2′-OH, a crucial question arises regarding whether a DNA-based probe, which lacks 2′-OH groups, can still form a kink-turn-like structure upon hybridization with the target RNA.

Unfortunately, despite multiple crystallization attempts, we could not obtain single crystals for DNA–2AP, preventing direct structural validation. Without this direct evidence, it remains unclear whether the DNA-based probe adopts a fully equivalent kink-turn structure. However, our fluorescence experiments with the DNA-based probe (Fig. 4) provide strong indirect evidence supporting the formation of a kink-turn-like conformation in which 2AP is fully bulged out. Specifically, the sequence-dependent fluorescence enhancement observed in DNA–2AP hybridization experiments suggests that the probe undergoes a structural rearrangement upon binding to the target RNA, mimicking the functional role of the kink-turn motif.

## Conclusion

In this study, we have succeeded in the structure-based design of a probe for sequence-specific RNA detection by mimicking the kink-turn motif found in various functional RNA molecules. This probe differs from conventional detection techniques based solely on base complementarity, such as molecular beacons. Instead, it is designed to form noncomplementary sheared G-A base pairs upon hybridization with the target RNA, leading to the formation of the kink-turn motif and subsequent fluorescence activation. The complementary sequence to the target RNA (RNA sequence recognition site) and the sequence necessary to form the kink-turn motif (functional site) are located at both ends and in the central part of the probe fragment, respectively. As long as the target RNA contains the sequence 5′-GA-3′, probe fragments can efficiently be designed to detect it in a sequence-specific manner. In the case of conventional detection techniques based solely on base complementarity, it often happens that the probe hybridizes to and detects unrelated RNAs that are similar in sequence to the target RNA. This is due to the fact that nucleic acids can form not only complementary base pairs such as A-T/A-U and G-C but also various noncomplementary base pairs [26]. The probe developed in this study is expected to be more accurate in RNA detection. The increase in fluorescence intensity is observed only when the probe fragment hybridizes with the target RNA by forming complementary base pairs and two alternative noncomplementary G-A base pairs, then correctly forming the kink-turn motif.

The kink-turn motif is found in various functional RNA molecules and is widely conserved from bacteria to archaea to eukaryotes, suggesting that it has excellent structural stability and homogeneity. In this study, we have confirmed that such RNA motifs, which have been preserved through natural selection, are highly suitable for structural biomimetics at the molecular level.

## Supplementary Material

ugaf006_Supplemental_File

## Data Availability

The crystal structures solved in this study were deposited in the Protein Data Bank (PDB ID: 7EI7, 7F8Z, 7EI9, and 7EIA).

## References

[1] Calin GA, Dumitru CD, Shimizu M et al. Frequent deletions and down-regulation of micro-RNA genes miR15 and miR16 at 13q14 in chronic lymphocytic leukemia. Proc Natl Acad Sci USA. 2002; 99:15524–9. 10.1073/pnas.242606799. 12434020 PMC137750

[2] Peng Y, Croce CM The role of microRNAs in human cancer. Sig Transduct Target Ther. 2016; 1:15004 10.1038/sigtrans.2015.4. PMC5661652 29263891

[3] Rao P, Benito E, Fischer A MicroRNAs as biomarkers for CNS disease. Front Mol Neurosci. 2013; 6:39 10.3389/fnmol.2013.00039. 24324397 PMC3840814

[4] Zhao Y, Jaber V, Alexandrov PN et al. MicroRNA-based biomarkers in Alzheimer’s disease (AD). Front Neurosci. 2020; 14:585432 10.3389/fnins.2020.585432. 33192270 PMC7664832

[5] Wang KC, Chang HY Molecular mechanisms of long noncoding RNAs. Mol Cell. 2011; 43:904–14. 10.1016/j.molcel.2011.08.018. 21925379 PMC3199020

[6] Schmitt AM, Chang HY Long noncoding RNAs in cancer pathways. Cancer Cell. 2016; 29:452–63. 10.1016/j.ccell.2016.03.010. 27070700 PMC4831138

[7] Tyagi S, Kramer FR Molecular beacons: probes that fluoresce upon hybridization. Nat Biotechnol. 1996; 14:303–8. 10.1038/nbt0396-303. 9630890

[8] Okamoto A ECHO probes: a concept of fluorescence control for practical nucleic acid sensing. Chem Soc Rev. 2011; 40:5815–28. 10.1039/c1cs15025a. 21660343

[9] Klein DJ, Schmeing TM, Moore PB et al. The kink-turn: a new RNA secondary structure motif. EMBO J. 2001; 20:4214–21. 10.1093/emboj/20.15.4214. 11483524 PMC149158

[10] Huang L, Lilley DMJ The kink turn, a key architectural element in RNA structure. J Mol Biol. 2016; 428:790–801. 10.1016/j.jmb.2015.09.026. 26522935 PMC5061560

[11] Huang L, Lilley DMJ The kink-turn in the structural biology of RNA. Quart Rev Biophys. 2018; 51:e5 10.1017/S0033583518000033. 30912490

[12] Matsumura S, Ikawa Y, Inoue T Biochemical characterization of the kink-turn RNA motif. Nucleic Acids Res. 2003; 31:5544–51. 10.1093/nar/gkg760. 14500816 PMC206460

[13] Ohno H, Kobayashi T, Kabata R et al. Synthetic RNA–protein complex shaped like an equilateral triangle. Nature Nanotech. 2011; 6:116–20. 10.1038/nnano.2010.268. 21240283

[14] Saito H, Kobayashi T, Hara T et al. Synthetic translational regulation by an L7Ae-kink-turn RNP switch. Nat Chem Biol. 2010; 6:71–8. 10.1038/nchembio.273. 20016495

[15] Huang L, Lilley DM The molecular recognition of kink-turn structure by the L7Ae class of proteins. RNA. 2013; 19:1703–10. 10.1261/rna.041517.113. 24149842 PMC3884654

[16] Kabsch W XDS. Acta Crystallogr D Biol Crystallogr. 2010; 66:125–32. 10.1107/S0907444909047337. 20124692 PMC2815665

[17] Zwart PH, Afonine PV, Grosse-Kunstleve RW et al. Automated structure solution with the PHENIX suite. Methods Mol Biol. 2008; 426:419–35. 10.1007/978-1-60327-058-8_28. 18542881

[18] McCoy AJ, Grosse-Kunstleve RW, Adams PD et al. Phaser crystallographic software. J Appl Crystallogr. 2007; 40:658–74. 10.1107/S0021889807021206. 19461840 PMC2483472

[19] Liebschner D, Afonine PV, Baker ML et al. Macromolecular structure determination using X-rays, neutrons and electrons: recent developments in Phenix. Acta Crystallogr D Struct Biol. 2019; 75:861–77. 10.1107/S2059798319011471. 31588918 PMC6778852

[20] Emsley P, Cowtan K Coot: model-building tools for molecular graphics. Acta Crystallogr D Biol Crystallogr. 2004; 60:2126–32. 10.1107/S0907444904019158. 15572765

[21] Emsley P, Lohkamp B, Scott WG et al. Features and development of Coot. Acta Crystallogr D Biol Crystallogr. 2010; 66:486–501. 10.1107/S0907444910007493. 20383002 PMC2852313

[22] Reuter JS, Mathews DH RNAstructure: software for RNA secondary structure prediction and analysis. BMC Bioinf. 2010; 11:129 10.1186/1471-2105-11-129. PMC2984261 20230624

[23] Tinsley RA, Walter NG Pyrrolo-C as a fluorescent probe for monitoring RNA secondary structure formation. RNA. 2006; 12:522–9. 10.1261/rna.2165806. 16431979 PMC1383589

[24] Xiong Y, Sundaralingam M Crystal structure and conformation of a DNA-RNA hybrid duplex with a polypurine RNA strand: d(TTCTTBr5CTTC)-r(GAAGAAGAA). Structure. 1998; 6:1493–501. 10.1016/S0969-2126(98)00148-8. 9862803

[25] Liu J, Lilley DMJ The role of specific 2'-hydroxyl groups in the stabilization of the folded conformation of kink-turn RNA. RNA. 2007; 13:200–10. 10.1261/rna.285707. 17158708 PMC1781366

[26] Leontis NB, Stombaugh J, Westhof E The non-Watson–Crick base pairs and their associated isostericity matrices. Nucleic Acids Res. 2002; 30:3497–531. 10.1093/nar/gkf481. 12177293 PMC134247

