# Supplementary material for: Structure-based design of a sequence-specific RNA probe that mimics the kink-turn motif
Source: NAR Mol Med. 2025 Mar 18;2(1):ugaf006. doi: 10.1093/narmme/ugaf006 (PMC12430006; doi:10.1093/narmme/ugaf006)
Supplement: ugaf006_Supplemental_File [file ugaf006_Supplemental_File.docx]

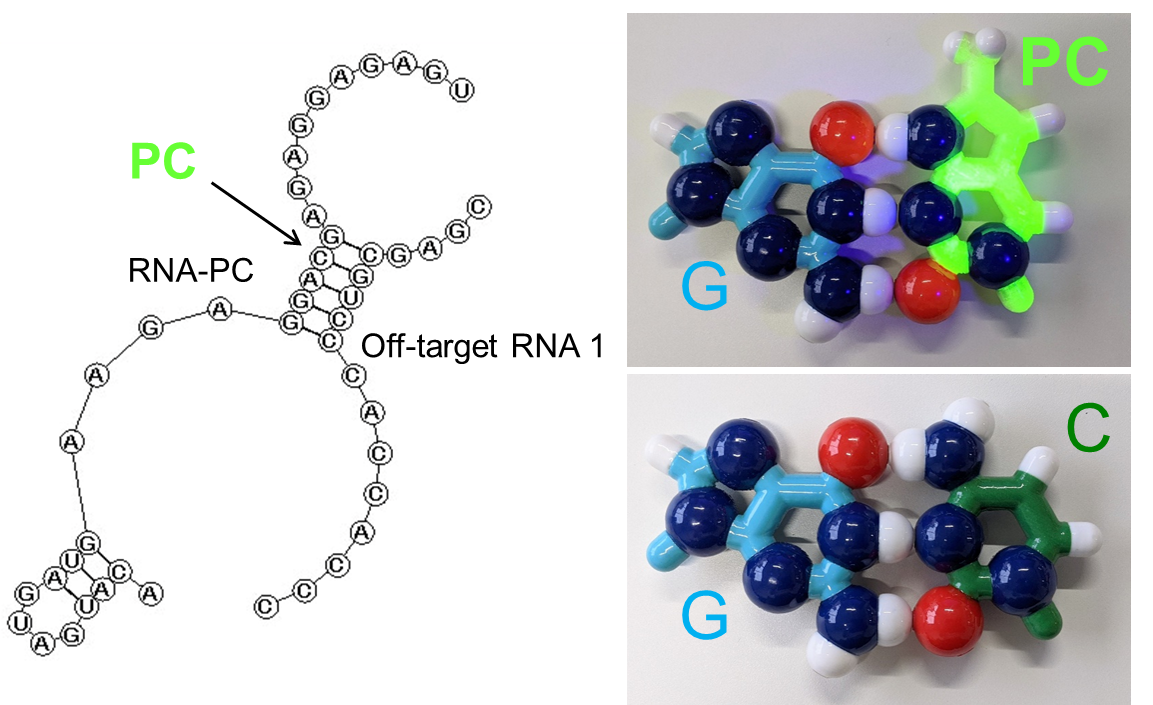


**Supplementary Figure 1.** Secondary structure prediction of the RNA-PC/Off-target RNA 1 complex estimated by using RNAstructrue. Pyrrolo-cytosine (PC) is capable of base pairing with guanine (G), similar to the Watson-Crick G-C base pair. The G-PC and G-C base pairs in this figure were constructed using the BasePairPuzzle (https://jkondo.wixsite.com/basepairpuzzle).


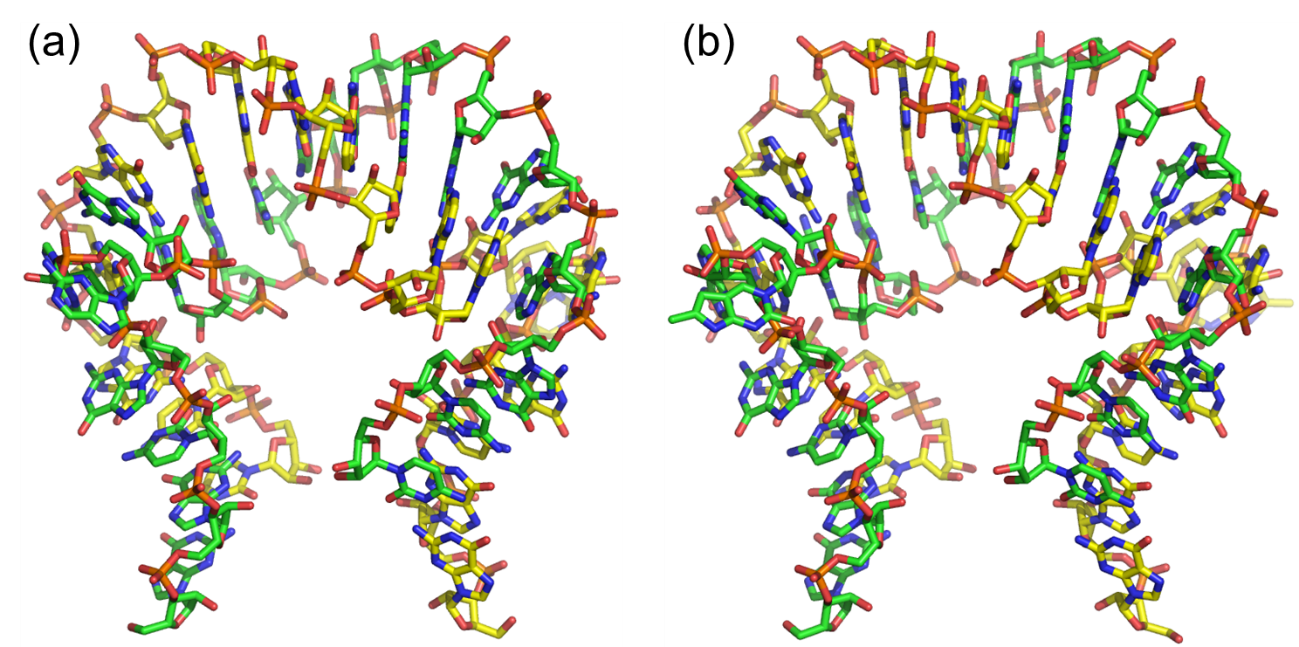


**Supplementary Figure 2.** Overall structures of Kt-2AP observed in the Kt-2AP-1 crystal (a) and Kt-PC observed in the Kt-PC-1 crystal (b).


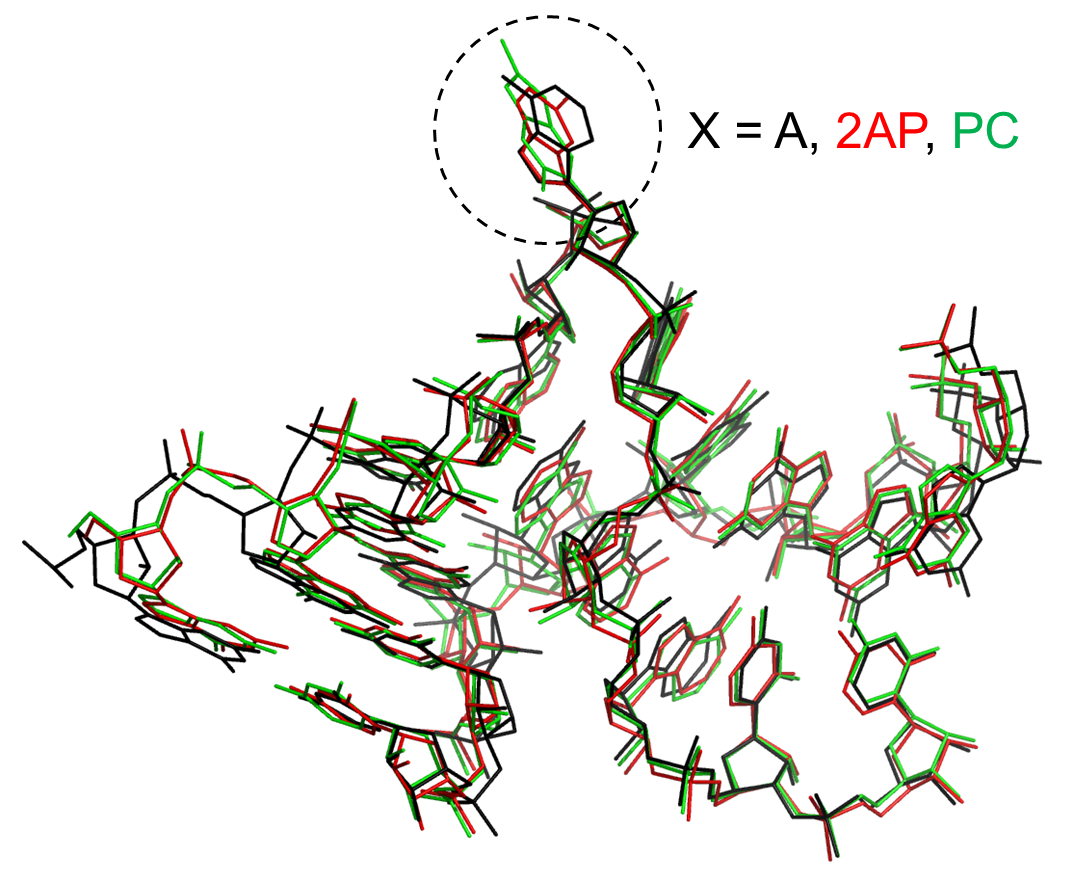


**Supplementary Figure 3.** Superposition of the crystal structures of Kt-2AP (red) and Kt-PC (green) onto the canonical kink-turn structure (black) (PDB ID: 4C40). The high degree of structural similarity confirms that the designed probes adopt the expected kink-turn conformation, supporting the validity of our fluorescence-based detection strategy."
